# Machine learning approach to predict protein phosphorylation sites by incorporating evolutionary information

**DOI:** 10.1186/1471-2105-11-273

**Published:** 2010-05-21

**Authors:** Ashis Kumer Biswas, Nasimul Noman, Abdur Rahman Sikder

**Affiliations:** 1 Department of Computer Science and Engineering, University of Dhaka, Dhaka - 1000, Bangladesh; 2 Center for Advanced Research in Chemical, Physical, Biological and Pharmaceutical Sciences, University of Dhaka, Dhaka - 1000, Bangladesh

## Abstract

**Background:**

Most of the existing *in silico *phosphorylation site prediction systems use machine learning approach that requires preparing a good set of classification data in order to build the classification knowledge. Furthermore, phosphorylation is catalyzed by kinase enzymes and hence the kinase information of the phosphorylated sites has been used as major classification data in most of the existing systems. Since the number of kinase annotations in protein sequences is far less than that of the proteins being sequenced to date, the prediction systems that use the information found from the small clique of kinase annotated proteins can not be considered as completely perfect for predicting outside the clique. Hence the systems are certainly not generalized. In this paper, a novel generalized prediction system, PPRED (**P**hosphorylation **PRED**ictor) is proposed that ignores the kinase information and only uses the evolutionary information of proteins for classifying phosphorylation sites.

**Results:**

Experimental results based on cross validations and an independent benchmark reveal the significance of using the evolutionary information alone to classify phosphorylation sites from protein sequences. The prediction performance of the proposed system is better than those of the existing prediction systems that also do not incorporate kinase information. The system is also comparable to systems that incorporate kinase information in predicting such sites.

**Conclusions:**

The approach presented in this paper provides an efficient way to identify phosphorylation sites in a given protein primary sequence that would be a valuable information for the molecular biologists working on protein phosphorylation sites and for bioinformaticians developing generalized prediction systems for the post translational modifications like phosphorylation or glycosylation. PPRED is publicly available at the URL http://www.cse.univdhaka.edu/~ashis/ppred/index.php.

## Background

One of the most critical cellular phenomenon is phosphorylation of proteins as it is involved in signal transduction of various processes including cell cycle, proliferation and apoptosis [1-3]. This phenomenon is catalyzed by protein kinases affecting certain acceptor residues (Serine, Threonine and Tyrosine) in substrate sequences. A study on 2D-gel electrophoresis showed that 30-50% of the proteins in an eukaryotic cell had undergone phosphorylation [4]. So, accurate prediction of the phosphorylation sites of eukaryotic proteins may help in understanding the overall intracellular activities.

Both experimental and computational methods have been developed to investigate the phosphorylation sites. But *in vivo *and *in vitro *methods are often time-consuming, expensive and have very limited scope due to some restrictions for many enzymatic reactions. On the other hand, *in silico *prediction of phosphorylation sites from computational approaches may provide fast and automatic annotations for candidate phosphorylation sites. Besides, there are web servers that provide experimental results of phosphorylation sites in proteins which were achieved after *in vivo *or *in vitro *experiments. For example, PHOSIDA [5] was developed as a phosphorylation site database which was integrated with thousands of high-confidence in vivo phosphorylated sites identified by mass spectrometry-based proteomics in five different species (*Homo sapiens*, *Mus musculus*, *Drosophila melanogaster*, *Caenorhabditis elegans *and *Saccharomyces cerevisiae*).

Whereas a range of *in silico *predictors have been developed using different machine learning techniques. For example, PPSP was developed applying Bayesian Decision Theory [6]. It can predict the phosphorylation sites for about 70 phospho-kinase groups. Training dataset of PPSP was collected from Phospho.ELM (version 2, September, 2004) [7] and the phosphorylation sites without kinase information were filtered out preserving only 1400 significant kinase specific phosphorylated sites. DISPHOS was developed using dataset from Swissprot with phosphorylation annotations on the eukaryotic proteins [8] resulting a total of 1500 such phosphorylation sites. In the prediction system called NetphosK, six Serine/Threonine kinases for which the largest number of known acceptor sites annotated in the phosphoBase [9] were identified. For each of the six kinases 22 to 258 different substrate sites were considered. Then the information derived from sequence logos of each of the groups were incorporated to train a neural network [10]. Kinasephos was developed using phosphoBase [9] and Swissprot(rel.45) protein dataset where only 1163 sites were found to have kinase annotations [11]. Several kinase groups were split into smaller subgroups using maximal dependence decomposition. Then each of the subgroups was separately used in the training phase to build profile Hidden Markov Model.

Scansite 2.0 identifies short sequence motifs that were recognized by phosphorylation on serine, threonine or tyrosine residues [12]. In this case, many of the motifs were determined using oriented peptide library experiments. The peptides that were phosphorylated by the kinase enzymes were isolated and sequenced as an ensemble by Edman degradation. When sequenced in this manner, each Edman cycle revealed the relative amount of each amino acid residue occurring at the corresponding positions. This information was scaled and normalized to get a type of PSSM (Position Specific Scoring Matrices). The PSSM, generated by this study did not include evolutionary information because it was based on a limited number of proteins that were phosphorylated by proteins with only same type of kinases. In such cases, evolutionary links between the protein under consideration with proteins without kinase annotations were not considered. NetPhos [13] is a neural network-based method for predicting potential phosphorylation sites at serine, threonine or tyrosine residues in protein sequences. This system did not consider any kinase specific information for prediction. The AutoMotif Server AMS [14] performs phosphorylation site predictions based only on local sequence information, for example- preferences of short segments around phosphorylation residues. This server also did not use kinase specific information during the training and the prediction phases. The group based prediction system, GPS [15] classified the protein kinases into a hierarchical structure with four levels, including group, family, subfamily and single protein kinase in the preparation of such prediction system.

The *in silico *prediction systems that included kinase information performed particularly well when kinase information of the target proteins was known or species or group specific classification knowledge was known beforehand. For example, experimental studies of phosphorylation in yeast revealed strong preferences for particular kinases for specific substrates and also indicated that predictions based on phosphorylation site patterns on those cases could lead to substantial over-prediction [16].

The current set of phosphorylation site prediction systems has recently been analyzed [17]. The analysis also revealed that the existing systems are not generalized in a sense that they were trained mainly with a limited number of proteins having kinase annotations that add noise in the performance of prediction systems when no kinase information is known. The prediction method (PPRED) proposed in this article moved ahead to overcome the limitation by incorporating only evolutionary information--PSSM profile of the proteins rather than using any kinase specific information. For a protein sequence, the PSSM profile, generated by PSI-BLAST (Position Specific Iterated Basic Local Alignment Search Tool) of NCBI describes the likelihood of a particular residue substitution at a specific position based on evolutionary information [18-20] and it provides more comprehensive information about proteins than a single sequence [21,22].

## Results and Discussion

### Cross Validation Performance

In the training dataset (namely *A*", collected from Phospho.ELM (ver. 8.1) [23]), there were 5724 phosphorylated proteins. The number of positive sites annotated by phospho.ELM and the number of negative sites annotated in our system for each of the three residues S (serine), T (Threonine) and Y (Tyrosine) are shown in Table 1. The PSSM profiles of the proteins of *A*" dataset provided the training instances for the SVMs. The ratio of the number of negative to positive sites was a big number which could definitely bias the SVMs training, that would lead in predicting most of the unknown sites as negative. Thus it was required to reduce the number of negative instances to overcome the problem. Four separate experiments were performed with training datasets containing number of positive to negative training instances having the ratios 1 : 2, 1 : 1 , 1 : 1 and 1 :  respectively.

**Table 1:** Number of sites of each of the three phosphorylated residues from dataset *A*".

Residue	Number of instances	
	
	Positive	Negative
S	12399	69446
T	2528	62302
Y	1829	43571

From this table it can be found that the number of negative sites greatly outnumbers that of the positive sites for each of the three residues.

In the first experiment, the training dataset was prepared to have the number of positive to negative training instances with ratio 1 : 2. To do this, the ratio  was first calculated, where *n *was the number of negative training instances and *p *was the number of positive training instances from the *A*" dataset. Then the expected training dataset was prepared by selecting every *r*^*th *^instance from the negative instance set. Then a three-fold cross validations were performed on this modified training dataset. Separate three fold cross validations were performed on the instance set for five different window sizes (7, 9, 11, 13 and 15) for each of the three residues (S, T and Y). Similarly in second, third and fourth experiment, the ratios were settled using the similar formulae: ,  and  respectively. Table 2, Table 3, Table 4 and Table 5 show the results of the cross validations using the aforementioned types of training datasets. Accuracy, Sensitivity, Specificity, Mcc and False Positive Rate (FPR) are also shown for each of the five different window sizes for each of the three residues.

**Table 2:** Three fold cross validation performance of the prediction system using ratio 1:2.

Residue	W	Ac(%)	Sn(%)	Sp(%)	Mcc	FPR
	7	78.47	29.57	95.94	0.36	0.04
	9	78.61	30.82	95.67	0.37	0.04
S	11	78.66	30.23	95.95	0.37	0.04
	13	78.78	31.53	95.66	0.38	0.04
	15	78.86	31.69	95.70	0.38	0.04

	7	74.92	34.02	94.84	0.38	0.05
	9	75.31	34.34	95.26	0.40	0.05
T	11	75.25	33.39	95.63	0.39	0.04
	13	75.30	32.76	96.01	0.40	0.04
	15	74.91	30.94	96.32	0.39	0.04

	7	71.90	12.13	99.50	0.27	0.01
	9	73.49	19.46	98.43	0.32	0.02
Y	11	72.94	17.11	98.71	0.31	0.01
	13	72.78	16.02	98.99	0.30	0.01
	15	72.40	14.21	99.27	0.29	0.01

The sensitivity (Sn) and the specificity (Sp) columns of the table reveal that the system using this ratio identifies most of the sites as negative.

**Table 3:** Three fold cross validation performance of the prediction system using ratio 1:1.5.

Residue	W	Ac(%)	Sn(%)	Sp(%)	Mcc	FPR
	7	74.94	51.80	87.33	0.43	0.13
	9	75.02	52.17	87.26	0.44	0.13
S	11	75.54	53.00	87.61	0.45	0.12
	13	75.78	53.46	87.73	0.45	0.12
	15	76.15	54.53	87.74	0.46	0.12

	7	72.44	46.76	89.11	0.41	0.11
	9	72.47	48.14	88.26	0.41	0.12
T	11	72.39	50.35	86.70	0.41	0.13
	13	72.70	50.47	87.13	0.42	0.13
	15	72.63	50.12	87.24	0.41	0.13

	7	70.38	36.69	91.60	0.36	0.08
	9	71.76	41.45	90.84	0.39	0.09
Y	11	71.84	41.12	91.19	0.39	0.09
	13	72.31	42.11	91.33	0.40	0.09
	15	72.12	40.74	91.88	0.39	0.08

Here in this table, the sensitivity (Sn) and the specificity (Sp) columns reveal that the system using this ratio identifies most of the sites as negative.

**Table 4:** Three fold cross validation performance of the prediction system using ratio 1:1.

Residue	W	Ac(%)	Sn(%)	Sp(%)	Mcc	FPR
	7	73.92	70.81	76.69	0.48	0.23
	9	74.19	70.02	77.92	0.48	0.22
S	11	74.41	70.16	78.20	0.49	0.22
	13	74.75	71.63	77.54	0.49	0.22
	15	74.83	73.12	76.35	0.50	0.24

	7	69.54	65.27	73.69	0.39	0.26
	9	69.71	66.49	72.84	0.40	0.27
T	11	70.18	67.52	72.76	0.41	0.27
	13	70.02	66.85	73.11	0.41	0.27
	15	70.22	68.31	72.07	0.41	0.28

	7	67.67	68.51	66.86	0.36	0.33
	9	68.74	67.80	69.66	0.38	0.30
Y	11	68.53	68.24	68.81	0.38	0.31
	13	68.45	68.07	68.81	0.37	0.31
	15	69.44	70.37	68.55	0.39	0.31

Using this ratio greatly balances the two accuracy parameters - Sensitivity and Specificity along with the Mathews correlation coefficient (Mcc). All the accuracy parameters show better performance if window size increases, i.e., more features are added to each of the training instances for the SVMs.

**Table 5:** Three fold cross validation performance of the prediction system using ratio 1:0.5.

Residue	W	Ac(%)	Sn(%)	Sp(%)	Mcc	FPR
	7	75.41	90.54	45.69	0.42	0.54
	9	75.61	89.35	48.62	0.43	0.51
S	11	76.45	89.65	50.52	0.45	0.49
	13	76.91	89.45	52.30	0.46	0.48
	15	77.21	90.03	52.04	0.47	0.48

	7	71.08	93.63	26.26	0.28	0.74
	9	70.89	92.40	28.14	0.28	0.72
T	11	71.58	93.24	28.54	0.30	0.71
	13	71.47	93.08	28.54	0.29	0.71
	15	71.45	93.59	27.44	0.29	0.73

	7	68.84	95.63	16.06	0.20	0.84
	9	70.58	94.26	23.93	0.27	0.76
Y	11	69.86	94.53	21.24	0.24	0.79
	13	69.57	94.21	21.02	0.23	0.79
	15	69.57	94.64	20.16	0.23	0.80

Here in this table, the sensitivity (Sn) and the specificity (Sp) columns reveal that the system using this ratio identifies most of the sites as positive.

From the results it can be observed that the PPRED showed optimum specificity and sensitivity if the sizes of the given positive and negative training datasets were equal. If the ratio was given less than 1, the sensitivity lowered but specificity rose, whereas if the ratio was given greater than 1, the opposite trend was observed.

#### Optimum choice of dataset ratio

From the experimental results shown in the Table 2, Table 3, Table 4 and Table 5 it is interesting to observe their ROC (Receiver Operating Characteristics) plot. We know that each discrete classifier produces a false positive rate, true positive rate pair that eventually corresponds to a single point in an ROC space [24].

**Figure 1:**
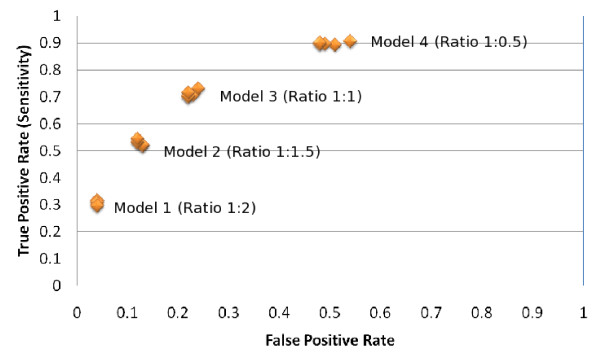
**ROC of the proposed prediction system for predicting serine sites**. There are five ROC points for each of the four models that represent the five window sizes (7,9,11,13 and 15) for the corresponding model.

**Figure 2:**
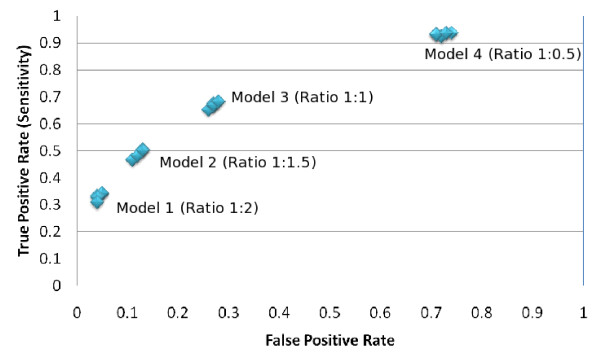
**ROC of the proposed prediction system for predicting threonine sites**. There are five ROC points for each of the four models that represent the five window sizes (7,9,11,13 and 15) for the corresponding model.

**Figure 3:**
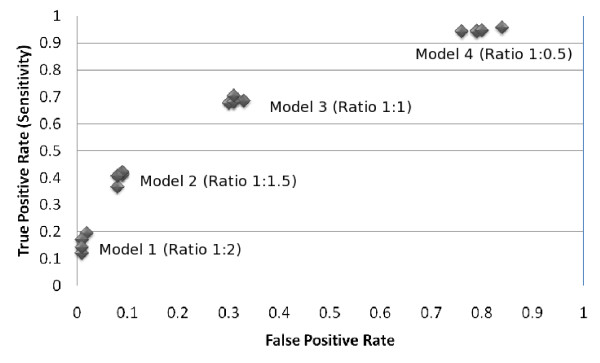
**ROC of the proposed prediction system for predicting tyrosine sites**. There are five ROC points for each of the four models that represent the five window sizes (7,9,11,13 and 15) for the corresponding model.

As our proposed system (PPRED) is a discrete classifier, it provides output which was only a class label (whether positive or negative). The ROC of each of the four experiments for each of the three residues (Serine, Threonine and Tyrosine) are shown in Figure 1, 2 and 3 respectively. It is to be noted that if the ratio of positive to negative training instances is chosen to be 1:1, all the assessment parameters show better figures. So the PPRED system will adhere to use the model 3 with ratio of the number of positive to negative instances to be 1:1.

#### Optimum choice of window size

The result shown in Table 4 reveals the fact that if more features were incorporated in a single instance of training data (that means increasing the window size), the prediction accuracy and sensitivity would increase. Nevertheless, there was a slight drop in the specificity.

But if the window size was increased beyond 15 (i.e., more features were added in an instance), the computational complexity and the required time for the SVMs training would increase exponentially. So the window size 15 can be considered as an optimum choice.

### Independent Benchmark Results

The independent benchmark dataset, named *B *in this study was collected from the article [17]. It contained 297 phospho-proteins and Table 6 shows the number of positive and negative sites in these proteins. The authors of that article randomly chose 400 phosphorylation sites from the Phospho.ELM [7] database (three hundreds from the version 6.0 and one hundred from the version 7.0 and Uniprot release 11.3). The PSSM profiles of the proteins from the *B *dataset were used to prepare testing instances that were given to the SVMs to assess the proposed prediction system using the classification knowledge built during the cross-validation phase. Like cross-validation phase, separate testing operations were performed on the testing instances for five different window sizes for each of the three residues as shown in Table 7. In predicting phospho-serine sites, PPRED showed accuracy of 61.34%, 67.82%, 68.44%, 67.77% and 64.96% for window sizes of 7, 9, 11, 13 and 15 respectively. Similar trend of increasing performance was found in the case of sensitivity, specificity and Mathews correlation coefficient parameters for increasing window size. So if a window size of 15 was chosen for S (serine), the system can achieve up to 65% accuracy, 72% sensitivity, 65% specificity with Mathews correlation coefficient to be 0.08. Almost similar observations were found in testing for T and Y sites. In case of T (Threonine) site prediction, the proposed system is 69.87% accurate, 67.06% sensitive, 69.90% specific with Mathews correlation coefficient of 0.07 with window size 15 and in case of Y site prediction with window size 15, it was found to be 65% accurate, 76% sensitive, 65% specific with the Mathews correlation coefficient 0.11. The independent benchmark test shown in Table 7 also underlines the importance of using more features (increased window size) to achieve better prediction performance.

**Table 6:** Number of sites of each of the three phosphorylated residues from dataset *B*.

Residue	Number of instances	
	
	Positive	Negative
S	923	3211
T	239	2897
Y	338	2120

This table also indicates that the number of negative sites outnumbered that of the positive sites.

**Table 7:** Prediction performance of the system when testing with the independent benchmark dataset.

Residue	W	Ac(%)	Sn(%)	Sp(%)	Mcc
	7	61.34	75.83	61.16	0.08
	9	67.82	67.77	67.82	0.08
S	11	68.44	70.14	68.42	0.09
	13	67.77	67.77	67.77	0.08
	15	64.96	72.04	64.88	0.08

	7	68.34	64.71	68.37	0.06
	9	73.81	58.82	73.93	0.07
T	11	71.73	61.18	71.81	0.06
	13	69.60	64.71	69.64	0.07
	15	69.87	67.06	69.90	0.07

	7	64.95	76.29	64.75	0.11
	9	62.40	79.38	62.09	0.11
Y	11	61.48	77.32	61.19	0.10
	13	61.88	80.41	61.54	0.11
	15	64.83	76.29	64.62	0.11

From this table it is evident that using ratio 1:1 shows good prediction performance for both positive and negative site predictions for each of the three residues. It is also evident that the performance increases if more features are included in each training instance (i.e., increasing the window size).

### Comparison with existing systems based on the benchmark

A random 297 protein entries were extracted from the Phospho.ELM database (ver. 6 and 7) with 211, 85 and 97 phosphorylated sites of serine, threonine and tyrosine respectively in the article [17] and the performance of the five existing prediction systems (PPSP [6], DISPHOS [8], KinasePhos [11], NetPhosK [10] and Scansite 2.0 [12]) were tested with these 393 annotations. But from Table 6, it can be found that more phosphorylation annotations were done by Phospho.ELM server (ver. 8.1), which became 923, 239 and 338 positive serine, threonine and tyrosine phosphorylated sites respectively on those 297 proteins. To compare the proposed system with the existing systems, the system was checked whether it can identify those 393 annotations.

Table 8 shows the comparison of the proposed system along with the nine existing prediction systems (PPSP [6], DISPHOS [8], KinasePhos [11], NetPhosK [10], Scansite 2.0 [12], AutoMotif Server AMS 2.0 [14], GPS 2.0 [15], PHOSIDA [5], NetPhos [13]) in terms of prediction scores (*Q*_3 _score), which is the number of correct identifications of phosphorylated sites. The window size was chosen to be 15 in PPRED that was found showing better prediction performance. The test result shows that the system can correctly predict 152, 57 and 74 phosphorylated serine, threonine and tyrosine sites out of 211, 85 and 97 annotated serine, threonine and tyrosine sites respectively of the independent benchmark.

**Table 8:** Comparison of the proposed system with existing nine prediction systems in terms of Q_3 _score according to Independent Benchmark.

Systems	Category	Prediction scores of the systems		
		
		S (%)	T (%)	Y (%)
KinasePhos [11]		83.9	88.2	85.6
NetPhosK [10]	Kinase Specific	90.5	84.7	53.6
PPSP [6]		98.6	92.9	90.7
GPS [15]		17.5	16.5	14.4

AutoMotif Server AMS [14]		64.5	62.4	54.6
DISPHOS [8]		96.7	96.5	90.7
NetPhos [13]		16.6	22.4	16.5
PHOSIDA [5]	Kinase Independent	8.5	1.2	3.1
Scansite [12]		29.9	18.8	35.1
PPRED (Proposed system)		72.0	67.1	76.3

There are a total of 211 phosphorylated serine sites, 85 threonine sites and 97 tyrosine sites in the benchmark dataset. Here it can be found easily that the proposed system (PPRED) shows better accuracy than AutoMotif Server AMS, GPS, NetPhos, PHOSIDA and Scansite 2.0 for predicting S, T and Y sites.

From the result it is evident that the proposed method has good prediction accuracy in predicting phosphorylated serine, threonine and tyrosine sites than those of AutoMotifServer AMS, GPS, NetPhos, PHOSIDA and Scansite 2.0.

Table 9, Table 10 and Table 11 show the detailed comparative analysis of the ten prediction systems including the proposed system (PPRED) in terms of serine, threonine and tyrosine site predictions respectively. Performance parameters, such as accuracy (Ac), sensitivity (Sn), specificity (Sp), Mathews correlation coefficient (Mcc) and False positive rate (FPR) are shown in the comparison tables. Each of the comparison tables underlines the competitive performance of the proposed system --PPRED among all other existing systems.

**Table 9:** Comparison of the proposed system with existing nine prediction systems in terms of Serine site prediction in the Independent dataset.

Systems	Category	Performance parameters of the systems				
		
		Ac(%)	Sn(%)	Sp(%)	Mcc	FPR(%)
KinasePhos [11]		93.11	9.48	94.13	0.02	5.87
NetPhosK [10]	Kinase Specific	85.24	14.22	86.11	0.00	13.89
PPSP [6]		80.24	17.54	81.00	-0.00	19.00
GPS [15]		80.31	17.54	81.08	-0.00	18.92

AutoMotif Server AMS [14]		36.25	64.45	35.90	0.00	64.10
DISPHOS [8]		89.40	14.69	90.31	0.02	9.69
NetPhos [13]		81.13	16.59	81.91	-0.00	18.09
PHOSIDA [5]	Kinase Independent	94.56	8.53	95.61	0.02	4.39
Scansite [12]		98.53	0.95	99.72	0.01	0.28
PPRED (Proposed system)		64.96	72.04	64.88	0.08	35.12

**Table 10:** Comparison of the proposed system with existing nine prediction systems in terms of Threonine site prediction in the Independent dataset.

Systems	Category	Performance parameters of the systems				
		
		Ac(%)	Sn(%)	Sp(%)	Mcc	FPR(%)
KinasePhos [11]		95.06	5.88	95.77	0.01	4.23
NetPhosK [10]	Kinase Specific	86.39	16.47	86.94	0.01	13.06
PPSP [6]		82.80	16.47	83.32	-0.00	16.68
GPS [15]		82.85	16.47	83.38	-0.00	16.62

AutoMotif Server AMS [14]		40.35	62.35	40.17	0.00	59.83
DISPHOS [8]		93.92	7.06	94.61	0.01	5.39
NetPhos [13]		82.19	22.35	82.66	0.01	17.34
PHOSIDA [5]	Kinase Independent	97.15	1.18	97.91	-0.01	2.09
Scansite [12]		98.93	0.00	99.71	-0.00	0.29
PPRED (Proposed system)		69.87	67.06	69.90	0.07	30.10

**Table 11:** Comparison of the proposed system with existing nine prediction systems in terms of Tyrosine site prediction in the independent dataset.

Systems	Category	Performance parameters of the systems				
		
		Ac(%)	Sn(%)	Sp(%)	Mcc	FPR(%)
KinasePhos [11]		94.39	2.06	96.06	-0.01	3.94
NetPhosK [10]	Kinase Specific	86.62	12.37	87.97	0.00	12.03
PPSP [6]		82.40	13.40	83.66	-0.01	16.34
GPS [15]		83.03	14.43	84.28	-0.00	15.72

AutoMotif Server AMS [14]		41.47	54.64	41.23	-0.01	58.77
DISPHOS [8]		94.07	8.25	95.63	0.02	4.37
NetPhos [13]		82.96	16.49	84.16	0.00	15.84
PHOSIDA [5]	Kinase Independent	97.18	3.09	98.89	0.02	1.11
Scansite [12]		97.37	3.09	99.08	0.03	0.92
PPRED (Proposed system)		64.83	76.29	64.62	0.11	35.38

## Discussion

Most of the existing phosphorylation site prediction systems use kinase specific information of the phosphorylated sites. In those cases, proteins without kinase annotations from the phosphorylation-positive dataset found to date from Phospho.ELM [23] or SWISS-PROT [25] were not considered and hence were filtered out in those systems. It can be found from the present update of Phospho.ELM dataset (August 12, 2008) that only 20% of the positive phosphorylation sites contain kinase annotations, that means more than 80% of the dataset are omitted in the design of the existing kinase specific prediction systems. These major truncations definitely ignore some important properties of phosphorylation sites, such as--evolutionary conservation of phospho-proteins. Our hypothesis is that this information would be useful in classifying phosphorylation sites. Moreover, this evolutionary conservation has been found useful in many other *in silico *prediction systems, such as, in the prediction of protein-protein interaction sites [26], prediction of DNA binding sites in proteins [27] or even finding motifs [28].

The outcome of this study was to give a direction in developing a phosphorylation site prediction system that uses the generalized information (such as evolutionary information) from all phosphorylated proteins rather than partial information obtained from the kinase-annotated proteins. It also directs that the evolutionary conservation can be a good candidate feature for the prediction purpose.

The proposed method (PPRED) was successful in overcoming the limitations of the kinase-specific prediction methods to separate the two classes of proteins --phosphorylated and non-phosphorylated proteins. In fact, our proposed system deliberately omitted the kinase specific information of the phosphorylation sites to underline the importance of the evolutionary profiles alone to predict phosphorylation sites. The prediction results also proved the hypothesis that the proposed system using only the evolutionary information of proteins can classify phosphorylated and non-phosphorylated sites from given primary sequences of protein accurately enough to be used compatibly with any existing system. In designing the prediction system all serine, threonine and tyrosine residues which were not annotated as phosphorylated and which were not positioned in the window of size 50 of any of the positive annotated residues were considered as negative phosphorylated sites. But some of the non-annotated sites, that were treated as negative sites in our study could be annotated as positive sites in future experiments, which would then require to re-train the whole system with new training data which will in turn increase the prediction accuracy.

It was found that the number of positive sites were far less than that of the negative sites. The number of negative sites adds bias to the assessment of the prediction accuracy. If all the positive and negative sites were used in the training dataset, experimental result would show most of the sites as negative. So to attain a good prediction accuracy, a reduction in negative training instances is required. But there is a debate on how much to reduce the number of negative instances. In this study separate experimental results enlightened that if the number of negative sites can be reduced in such a way that the number becomes equal to that of positive sites, the prediction system shows its best performance.

Furthermore, the number of sites in serine, threonine and tyrosine were not also equal. So three separate prediction modules were built in the proposed PPRED system for detecting the probable sites of the three phosphorylated residues (S, T and Y). For example, whenever a serine site is to be predicted for phosphorylation event, concerned module takes over the job which actually overcome the problem of biasing by number of phosphorylated sites of other residues (in this case, T and/or Y).

Experimental results (Table 8) showed that the prediction score of the proposed system (PPRED) exhibit better performance in predicting phosphorylated sites than those of the AutoMotif Server AMS, GPS, NetPhos, PHOSIDA and Scansite 2.0 systems. Again, the PPRED uses only the evolutionary information of proteins in classification, whereas other existing methods --KinasePhos, NetPhosK, PPSP and DISPHOS used either kinase group information or many other features to train their corresponding machine learning programs. In this direction, performance of the PPRED is comparable to those prediction systems (Table 8).

The results shown in Table 9, Table 10 and Table 11 established the fact that evolutionary information has a good relation with the protein phosphorylation and hence can contribute in designing a good prediction system.

## Conclusions

In this work, a novel phosphorylation site prediction system, PPRED was presented that incorporated only the evolutionary information of the proteins of both phosphorylated and non-phosphorylated classes. Experimental results of the system revealed that the system exhibits better prediction performance than some of the existing kinase specific and non-specific prediction systems. The results of the experiments also underlined the significance of using evolutionary information of both phosphorylated and non-phosphorylated proteins which were used as the only classification feature in the proposed system. Comparing the proposed system with other approaches, it was found that the proposed method provides a generalized and a more consistent prediction performance in all the cases. The incorporation of the evolutionary information contributed in both classifying the two types of sites and making the system more generalized.

## Methods

### Prediction System Design

**Figure 4:**
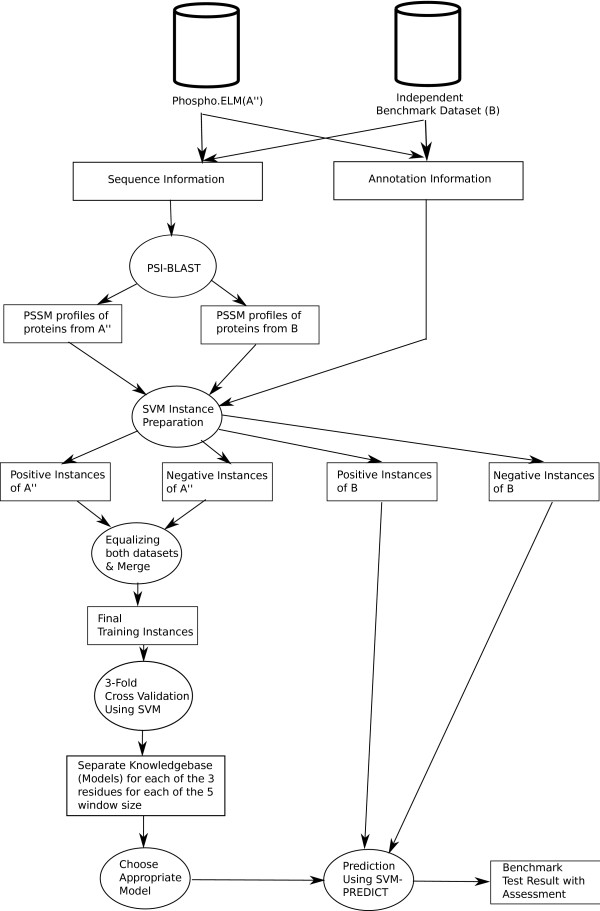
**Detailed system flow of the proposed prediction system during the test phase**.

The work flow for testing the proposed system with the independent benchmark dataset (*B*) is shown in Figure 4.

From the work flow diagram, sequences of both *A" *and *B *datasets were given to PSI-BLAST's "blastpgp" program to generate the PSSM profiles, which are the encapsulated representation of the evolutionary information of the proteins. The SVMs training instances were then prepared from the PSSM profiles. There were two classes of instances for both of the datasets *A" *and *B*. The positive and the negative class instances of *A" *were equalized in terms of number of instances and both were merged together to prepare the training set.

A three-fold cross validation was performed on the final merged instance set using the SVMs training module. Separate model files (Knowledge base file) for each of the three phosphorylated residues (S, T and Y) and for each of the five different window sizes (7, 9, 11, 13 and 15) were stored on the disk. Each of the individual cross validation results were reported in the result section.

To test the system, the instance set *B *was used and an appropriate model file stored in the cross-validation phase was chosen by looking at the type of residue and size of the window. The chosen model file and instance set *B *were given to the SVMs prediction module for testing. The SVMs prediction module performs predictions of phosphorylation sites on the given instance set from *B *based on the given classification knowledge Base (model) file.

**Figure 5:**
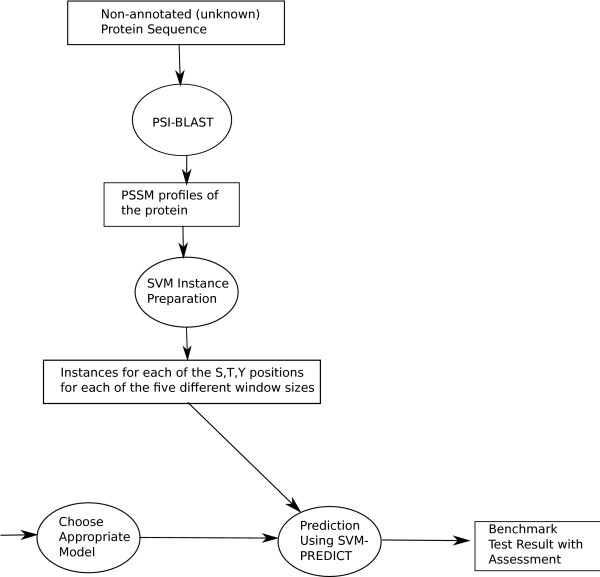
**System flow of the prediction system for predicting the phosphorylation sites for a given unknown protein sequence**.

Figure 5 illustrates the flow of operations of the PPRED system when an unknown protein sequence is given to it for prediction. Firstly the PSI-BLAST was employed to generate the PSSM profile of the given protein sequence. Then separate SVMs testing instances were prepared for each of the three residues (S, T and Y) and for each of the five different window sizes (7, 9, 11, 13 and 15). Appropriate knowledge-base (model) file is chosen that was stored at the cross-validation phase to predict "target labels" (+1:Positive, -1:Negative) for each of the testing instances.

The following sections explain each of the essential components of the PPRED system.

### Evolutionary Information of Proteins

The proposed method incorporates evolutionary information of phosphorylation sites. If we perform a multiple sequence alignment of the proteins against an *nr *(non-redundant) dataset of proteins, we will get a score of each of the twenty amino acids against each position of the target protein. The scores represent the evolutionary conservation information among the members of its lineage. This information can be represented as a two dimensional matrix which is known as the PSSM profile of the protein.

It was observed in this study that PSSM scores across a predefined window of a phosphorylated residue of some protein sequences have similar lineage of evolution. Hence the scores obtained from the PSSM profiles of phosphorylated proteins across a predefined window can be a good source of classification data for a prediction system. The PSSM profile of phosphorylated proteins were generated using PSI-BLAST method [18-20].

### Dataset Preparation

Two sources of dataset were used in this study. The first dataset is the Phospho.ELM version 8.1 that was released on August 12, 2008 [23] and was named *A *dataset in this study. The *A *dataset contains 6019 protein entries with a total of 18253 annotations of phosphorylation sites. Of these annotations 13320 phosphorylated serine sites, 2766 threonine sites, 2166 tyrosine sites were annotated (Additional file 1). There was an annotation of phosphorylated histidine which was discarded from this experiment because the objective of this work is to classify only the most frequently occurred phosphorylated residues which are serine, threonine and tyrosine residues. So, a new dataset *A*' was prepared from the dataset *A *that contained every proteins of the *A *dataset except the protein containing the histidine phosphorylated site (Additional file 2).

The second dataset was collected from the article [17] which they used in assessing the performance of some existing prediction systems. This independent benchmark dataset was named *B *dataset in our study. The *B *dataset contains 297 protein entries with annotations of 211 serine, 85 threonine and 97 tyrosine phosphorylated sites. But the *B *dataset contained 294 protein entries which were also in the *A*' dataset, so these common 294 protein entries were discarded from the *A*' dataset to form a new training dataset *A" *which is disjoint from the testing dataset *B*. The *A" *dataset and the independent benchmark dataset *B *can be found in the Additional file 3 and Additional file 4 respectively.

#### Positive Dataset Preparation

PSSM profiles of all the proteins of *A" *and *B *datasets were generated using PSI-BLAST search against the non-redundant (*nr*) database of protein sequences. A PSSM matrix for each of the proteins was generated by the "blastpgp" program of the PSI-BLAST package with three iterations of searching at cutoff *E*-value of 0.001 for inclusion of sequences in subsequent iterations.

For example, the command to generate a PSSM profile of the protein with accession P16386 is given below:

blastpgp   -d nr   -i "P16386.seq"   -j 3   -h 0.001   -Q "P16386.pssm"

Here, "P16386.seq" file contains the primary sequence of the protein P16386 in raw format. The option "-j 3" is to run "blastpgp" program for three iterations. The option "-h 0.001" is to restrict including unrelated sequences with a cutoff *E*-value of 0.001. The "-Q" option redirects the resultant PSSM profile to be saved in a file named "P16386.pssm".

**Figure 6:**
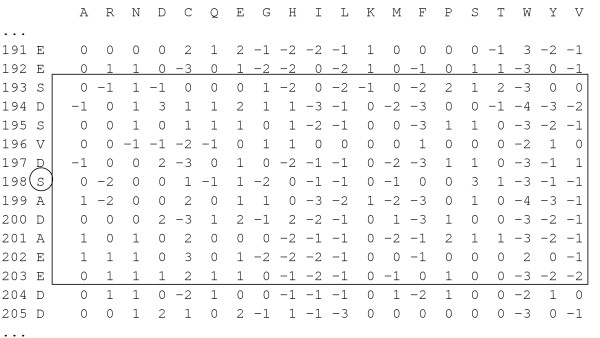
**Fragment of the PSSM Profile of the protein P12023 to form a training instance for the SVMs**. The fragment of this PSSM Profile of the protein P12023 includes position specific scores from position 191 to 205. Here, serine at position 198 is non-phosphorylated (according to the rule of negative annotations in our study) and hence the linear arrangement of the scores 5 positions upstream and 5 positions downstream from the position 198 form a negative instance of window size 11. A negative instance of serine residue was prepared by choosing a window over the non-phosphorylated serine of size 11 and convert the 11 × 20 matrix into a 220 size single dimensional linear array of scores which will eventually represent a negative instance for the SVMs-training.

The PSSM thus generated contained the probability of occurrence of each type of amino acid at each position. The evolutionary information for each amino acid is encapsulated in a vector of *L *× 20 dimensional matrix, where *L *is the length of the given protein sequence. Figure 6 demonstrates a fragment of PSSM profile of a protein with window size 11.

#### Negative Dataset Preparation

The most significant problem while compiling datasets for machine learning is that there is no negative data included in any of the known databases. Knowing the fact that a specific serine or threonine or tyrosine is not phosphorylated is extremely useful when designing a binary prediction method. Unfortunately, such information is very rarely published. To conclusively prove that a site is negative under all conditions is impossible.

In this study the non-annotated sites satisfying the criteria stated in the Proposition 1 were considered as negative sites.

**Proposition 1**. *A non-annotated residue is considered as negative site if it is not in a distance of 50 residues from any phosphorylation annotated residue of a protein sequence*.

Unfortunately, some of the The negative sites obtained using the Proposition 1 could be proved to be positive in future experiments. However, negative sites were used in this study because only a few serine, threonine and tyrosine residues are phosphorylated and the PSSM profile scores of the phosphorylated serine, threonine and tyrosine residues is skewed away from that of the scores of non-phosphorylated serine, threonine and tyrosine residues. Therefore, SVMs, which in practice allow some training errors, would regard false negative sites as errors.

The same approach of generating positive features of serine, threonine and tyrosine was employed to generate negative features as well.

### Support Vector Machines

The Support Vector Machines (SVMs) is a supervised learning algorithm for two-group classification problems [29,30]. The SVMs is known for its high performance in classifying unknown data and has been applied to many problem areas. The SVMs map the feature vector into a high dimensional feature space and classifies the samples by separating the hyper-plane in the space. At the training stage, SVMs search for an optimal hyper-plane by solving a quadratic programming optimization problem. This hyper-plane, determined by the criterion that maximizes the distance of nearest feature vector, has good generalization performance. We used LIBSVM (Library for Support Vector Machines) [31], with a radial basis function (RBF) kernel to predict phosphorylation sites. The SVMs using the RBF kernel has two parameters, γ and *C*. We fixed *C *and γ at default values of 1 and  respectively, where *k *is the number of attributes (features) in each instance of training dataset.

### Training System Design

From the PSSM profiles of the proteins of the *A" *dataset, negative and positive instances were prepared for each of the three phosphorylated residues --S, T and Y. The number of training instances of each residue of certain label (positive or negative) are shown in Table 1.

As mentioned above that a subset of non-annotated phosphorylation sites in the dataset *A" *is used as negative dataset. But from Table 1 it is evident that, the size of negative dataset greatly outnumbered that of the positive dataset for each of the three residues. If the SVMs were trained with these positive and negative datasets, it would predict most of the sites as negative [32].

To overcome this problem, reduction of size of the negative dataset was necessary. This study performs four separate experiments that reduces the size of negative dataset to become twice, one and a half times, half times and equal to that of the positive dataset. For example, to prepare the training dataset to have the same number of instances of positive and negative labels, the ratio *r *of negative dataset size to positive dataset size was first calculated. Then the positive dataset was kept intact (as the number of positive instances is less than that of negative instances), and all but every *r*^*th *^instance of the negative dataset were truncated. This way, the size of the negative dataset was made equal to the size of the positive dataset. After this equalizing, both the positive and the negative dataset were merged together to form the training dataset for the SVMs. In the other three experiments similar technique was employed to prepare the training datasets for the tree other different ratios. The detail results of the four experiments were discussed in the subsection "Cross Validation Performance" under the section "Results and Discussion".

### Testing the Proposed System

To evaluate the performance of the system two separate testing phases were performed. In the first phase, a 3-fold cross validation was used and in the second phase, dataset *B *was used.

• **Phase 1: Three fold cross validation test **In the three-fold cross validation, the merged training dataset was divided into three equal sets. Of these three sets, two sets were used for training and the remaining set was used for testing. This process was repeated three times in such a way that each of the three sets is used once for testing. The final performance parameters were obtained by averaging the performance of all the three sets. It should be noted that, in each of the three training phases, the SVMs produced a knowledge base (Model File), which were stored on disk and used later during prediction.

• **Phase 2: Prediction system Testing with Benchmark dataset **In the second phase of the assessment, the *B *dataset was used as a testing dataset and the system predicted this dataset using the stored knowledge base obtained in the cross validation phase.

### Prediction System Assessment

Most of the prediction systems are assessed by measuring the accuracy (*Ac*), sensitivity (*Sn*), specificity (*Sp*) and Matthews correlation coefficient (*Mcc*). However, when the accuracy of the positive predictions and those of the negative predictions are considered simultaneously, *Sn *and *Sp *values are both inadequate. Furthermore, if the numbers of both classes are different, the *Ac *value - the measurement that considers only the number of correct predictions - is not useful either. In addition to *Ac*, *Sn*, *Sn *values, *Mcc *can also be used to assess a prediction system. The *Mcc *has a value ranging from -1 to +1. The closer the *Mcc *value is to +1, the better the prediction system. We have used another parameter *FPR *(False Positive rate) to draw the ROC plot of the proposed system. If *Sn*, *Sp, Ac*, *Mcc *and *FPR *are each expressed in terms of true positive (*TP*), false positive (*FP*), true negative (*TN*) and false negative (*FN*), each measurement is given shown below:

## Availability and Requirements

The PPRED (**P**hosphorylation **Pred**ictor) web server is publicly accessible at the URL http://www.cse.univdhaka.edu/~ashis/ppred/index.php. An internet browser is all that is needed to use the server. Any protein sequence of length at most 10000 residues (in FASTA or raw sequence format) can be submitted along with the submitter's email address to the PPRED server. The PPRED server then performs the prediction task and notifies the submitter of the task through his or her email within a short period of time. The PPRED web server is installed in a desktop computer assembled with an Intel(R) Pentium(R) 4 CPU 2.40 GHz (family 15, stepping 7) with Level 2 cache size of 512 Kilo Bytes and 512 Mega Bytes of DDR 1 RAM. The PPRED server program is installed on Fedora core 9 operating system. The installed softwares are: LIBSVM version 2.9 and BLAST version 2.2.19 (with NR database release of Nov 16, 2008). All training and testing datasets can be found in downloadable format at that URL. It is worth mentioning that the time required to predict phosphorylation sites from a given protein sequence depends on the length of the protein sequence.

## Abbreviations

**PSSM**: Position Specific Scoring Matrices; BLAST: Basic Local Alignment Search Tool; PSI-BLAST: Position Specific Iterated BLAST; NCBI: National Center for Biotechnology Information; SVMs: Support Vector Machines; S: Serine; T: Threonine; Y: Tyrosine; ROC: Receiver operating characteristic; RBF: Radial Basis Function.

## Authors' contributions

AKB carried out the study relating to evolutionary information of proteins, participated in the sequence alignment to extract evolutionary information and drafted the manuscript. NN participated in the design of the study and supervised the whole research work. ARS participated in the design of the support vector features from the evolutionary information of proteins. All authors have read, revised and approved the final manuscript.

## Supplementary Material

Additional file 1 **Training dataset: *A***. The A dataset is the phospho.ELM version 8.1 database that was released on Aug 12, 2008. The dataset contains 6019 protein entries with a total of 18253 annotations of phosphorylation sites. Of these annotations 13320 phosphorylated serine sites, 2766 threonine sites, 2166 tyrosine sites were annotated. There was an additional annotation of phosphorylated histidine. The Phospho.ELM is a database of S/T/Y phosphorylation sites hosted at the URL http://phospho.elm.eu.org/. It was collected on December 2008. Click here for file

Additional file 2 **Training dataset: *A'***. dataset was prepared from the dataset *A *that contained every proteins of the *A *dataset except the protein containing the histidine phosphorylated site. We discarded this entry because the objective of this work was to classify only the most frequently occurred phosphorylated sites which are serine, threonine and tyrosine residues. Click here for file

Additional file 3 **Training dataset: *A"***. The testing dataset *B *contains 294 protein entries which were also in the *A*' dataset. So these common 294 entries were discarded from the *A*' dataset to form a new training dataset *A" *which is disjoint from the testing dataset *B*. Click here for file

Additional file 4 **Testing dataset: *B *(Independent benchmark dataset)**. The *B *dataset was collected from the article [17] which the authors of that article used in assessing the prediction performance of the existing five prediction systems. This independent dataset was considered as *B *dataset in our study. The *B *dataset contains 297 protein entries, with a total of 393 annotations of phosphorylated serine, threonine and tyrosine sites. Of these 211 serine, 85 threonine and 97 tyrosine sites were annotated. Click here for file

## References

[B1] Cohen P The origins of protein phosphorylation Nat Cell Biol 2002 4 5 E127 30 10.1038/ncb0502-e127 11988757

[B2] Lawlor M Alessi D PKB/Akt a key mediator of cell proliferation, survival and insulin responses? Journal of Cell Science 2001 114 16 2903 2910 11686294 10.1242/jcs.114.16.2903

[B3] Hunter T The Croonian Lecture 1997. The phosphorylation of proteins on tyrosine: its role in cell growth and disease Philosophical Transactions of the Royal Society B: Biological Sciences 1998 353 1368 583 10.1098/rstb.1998.0228 PMC1692245 9602534

[B4] Pinna L Ruzzene M How do protein kinases recognize their substrates? BBA-Molecular Cell Research 1996 1314 3 191 225 8982275 10.1016/s0167-4889(96)00083-3

[B5] Gnad F Ren S Cox J Olsen J Macek B Oroshi M Mann M PHOSIDA (phosphorylation site database): management, structural and evolutionary investigation, and prediction of phosphosites Genome biology 2007 8 11 R250 10.1186/gb-2007-8-11-r250 18039369 PMC2258193

[B6] Xue Y Li A Wang L Feng H Yao X PPSP: prediction of PK-specific phosphorylation site with Bayesian decision theory BMC Bioinformatics 2006 7 163 10.1186/1471-2105-7-163 16549034 PMC1435943

[B7] Diella F Cameron S Gemünd C Linding R Via A Kuster B Sicheritz-Pontén T Blom N Gibson T Phospho.ELM: a database of experimentally verified phosphorylation sites in eukaryotic proteins BMC bioinformatics 2004 5 79 10.1186/1471-2105-5-79 15212693 PMC449700

[B8] Iakoucheva L Radivojac P Brown C O'Connor T Sikes J Obradovic Z Dunker A The importance of intrinsic disorder for protein phosphorylation Nucleic acids research 2004 32 3 1037 10.1093/nar/gkh253 14960716 PMC373391

[B9] Kreegipuu A Blom N Brunak S PhosphoBase, a database of phosphorylation sites: release 2.0 Nucleic Acids Research 1999 27 237 239 10.1093/nar/27.1.237 9847189 PMC148144

[B10] Blom N Sicheritz-ponten T Gupta R Gammeltoft S Brunak S Prediction of post-translational glycosylation and phosphorylation of proteins from the amino acid sequence Proteomics(Weinheim. Print) 2004 4 6 1633 1649 10.1002/pmic.200300771 15174133

[B11] Huang H Lee T Tzeng S Horng J KinasePhos: a web tool for identifying protein kinase-specific phosphorylation sites Nucleic Acids Research 2005 33 W226 10.1093/nar/gki471 15980458 PMC1160232

[B12] Obenauer J Cantley L Yaffe M Scansite 2.0: proteome-wide prediction of cell signaling interactions using short sequence motifs. Article Nucleic Acids Research 2003 31 13 3635 3641 10.1093/nar/gkg584 12824383 PMC168990

[B13] Blom N Gammeltoft S Brunak S Sequence and structure-based prediction of eukaryotic protein phosphorylation sites Journal of Molecular Biology 1999 294 5 1351 1362 10.1006/jmbi.1999.3310 10600390

[B14] Plewczynski D Tkacz A Wyrwicz L Rychlewski L AutoMotif server: prediction of single residue post-translational modifications in proteins Bioinformatics 2005 21 10 2525 10.1093/bioinformatics/bti333 15728119

[B15] Xue Y Ren J Gao X Jin C Wen L Yao X GPS 2.0, a tool to predict kinase-specific phosphorylation sites in hierarchy Molecular & Cellular Proteomics 2008 7 9 1598 10.1074/mcp.M700574-MCP200 PMC2528073 18463090

[B16] Ptacek J Devgan G Michaud G Zhu H Zhu X Fasolo J Guo H Jona G Breitkreutz A Sopko R Global analysis of protein phosphorylation in yeast Nature 2005 438 7068 679 684 10.1038/nature04187 16319894

[B17] Sikder AR Zomaya AY Analysis of protein phosphorylation site predictors with an independent dataset International Journal of Bioinformatics Research and Applications 2009 5 20 37 10.1504/IJBRA.2009.022461 19136362

[B18] Altschul S Madden T Schaffer A Zhang J Zhang Z Miller W Lipman D Gapped BLAST and PSI-BLAST: a new generation of protein database search programs Nucleic acids research 1997 25 17 3389 10.1093/nar/25.17.3389 9254694 PMC146917

[B19] Altschul S Wootton J Gertz E Agarwala R Morgulis A Schäffer A Yu Y Protein database searches using compositionally adjusted substitution matrices The FEBS journal 2005 272 20 5101 10.1111/j.1742-4658.2005.04945.x 16218944 PMC1343503

[B20] Schaffer A Aravind L Madden T Shavirin S Spouge J Wolf Y Koonin E Altschul S Improving the accuracy of PSI-BLAST protein database searches with composition-based statistics and other refinements Nucleic Acids Research 2001 29 14 2994 10.1093/nar/29.14.2994 11452024 PMC55814

[B21] Kaur H Raghava G Prediction of *β*-turns in proteins from multiple alignment using neural network Protein Science 2003 12 3 627 634 10.1110/ps.0228903 12592033 PMC2312433

[B22] Kaur H Raghava G Prediction of-Turns in Proteins Using PSI-BLAST Profiles and Secondary Structure Information Proteins: Structure, Function, and Bioinformatics 2004 55 83 90 10.1002/prot.10569 14997542

[B23] Diella F Gould C Chica C Via A Gibson T Phospho.ELM: a database of phosphorylation sites update 2008 Nucleic Acids Research 2008 36 Database D240 D244 17962309 10.1093/nar/gkm772 PMC2238828

[B24] Fawcett T An introduction to ROC analysis Pattern recognition letters 2006 27 8 861 874 10.1016/j.patrec.2005.10.010

[B25] Boeckmann B Bairoch A Apweiler R Blatter M Estreicher A Gasteiger E Martin M Michoud K O'Donovan C Phan I The SWISS-PROT protein knowledgebase and its supplement TrEMBL in 2003 Nucleic Acids Research 2003 31 365 10.1093/nar/gkg095 12520024 PMC165542

[B26] Kakuta M Nakamura S Shimizu K Prediction of Protein-Protein Interaction Sites Using Only Sequence Information and Using Both Sequence and Structural Information Information and Media Technologies 2008 3 2 351 361

[B27] Ahmad S Sarai A PSSM-based prediction of DNA binding sites in proteins BMC bioinformatics 2005 6 33 10.1186/1471-2105-6-33 15720719 PMC550660

[B28] Hertzberg L Zuk O Getz G Domany E Finding motifs in promoter regions Journal of Computational Biology 2005 12 3 314 330 10.1089/cmb.2005.12.314 15857245

[B29] Cortes C Vapnik V Support-vector networks Machine Learning 1995 20 3 273 297

[B30] Vapnik V Statistical learning theory 1998 John Wiley & Sons, New York

[B31] Chih-Chung Chang Chih-Jen Lin LIBSVM: a library for support vector machines 2001 http://www.csie.ntu.edu.tw/~cjlin/libsvm/

[B32] Fan R Chen P Lin C Working set selection using second order information for training support vector machines The Journal of Machine Learning Research 2005 6 1889 1918

